# Variation in the magnitude of morphological and dietary differences between individuals among populations of small benthic Arctic charr in relation to ecological factors

**DOI:** 10.1002/ece3.3761

**Published:** 2018-01-03

**Authors:** Bjarni K. Kristjánsson, Camille A. Leblanc

**Affiliations:** ^1^ Hólar University College Sauðárkrókur Iceland

**Keywords:** habitat type, Iceland, pairwise diet similarity, pairwise Procustes distances, temperature

## Abstract

The early stages of intraspecific diversity are important for the evolution of diversification and speciation. Early stages of diversification can be seen in individual specialization, where individuals consume only a portion of the diet of the population as a whole, and how such specialization is related to phenotypic diversity within populations. Here, we study the strength of the relationship between morphological and dietary distances among individuals in eighteen populations of Icelandic small benthic charr. We furthermore studied if the strength of the relationship could be related to variation in local ecological factors these populations inhabit. In all the populations studied, there was a clear relationship between morphological and dietary distances, indicating that fish that had similar morphology were at the same time‐consuming similar food items. Our findings show a systematic variation in the relationship between morphology and diet at early stages of diversification in a highly specialized small benthic charr morph. The results show the importance of fine scale comparisons within populations and furthermore the value that systematic comparisons among populations under parallel evolution can contribute toward our increased understanding of evolutionary and ecological processes.

## 1 INTRODUCTION

The understanding of intraspecific diversity, both among and within populations, is a key focus of evolutionary studies. It is now accepted that ecological and evolutionary processes may interplay at a contemporary timescale to promote the emergence and/or maintenance of such biodiversity (Caroll, Hendry, Reznick, & Fox, 2007; Pelletier, Garant, & Hendry, 2009; Post & Palkovacs, 2009). Most commonly, studies have focused on population divergence, leading to morph formation and/or sympatric speciation (Grant & Grant, 2002; Schluter, 2000), with the majority of studies focusing on the later stages of divergence, for example, ecological speciation (Schluter, 2000) and/or adaptive radiation within species (Bolnick, 2006).

The early stages of divergence within a single population have been comparatively less well studied. Commonly, animal populations that were considered to display a generalist strategy were later found to be a collection of relatively specialized individuals (Bolnick et al., 2003). Such specialization and diversity among individuals can be looked at as the lowest level of intraspecific divergence within a single population, where higher levels include resource‐morph formation (Skúlason & Smith, 1995), and ultimately ecological speciation (Schluter, 2000). Populations within the same species, or related species, can be found at different levels of this speciation continuum (Hendry et al. 2009), and the levels are not necessarily stable through time, that is, change can happen quite fast (Hendry & Kinnison, 1999; Seehausen, Takimoto, Roy, & Jokella, 2008). In fact, it is important to study the lowest level of divergence such as the evolution of individual specialization because it may result in increased phenotypic diversity (Svanbäck & Bolnick, 2005) which may in turn promote further population divergence (Svanbäck & Bolnick, 2005, 2007), for example, through frequency‐dependent interactions that may underlie disruptive selection (Bürger & Gimelfarb, 2004; Dieckmann & Doebeli, 1999; Svanbäck & Bolnick, 2007). Yet, how key (ecological) factors shape this lowest level of divergence is poorly understood.

Individual diet specialization can be defined as the proportion of the diet of an individual relative to the diet of the population (Araujo, Bolnick, & Layman, 2011). Individual specialization constitutes one of the finest scales of diversity because it characterizes resource use at the individual level. By developing a quantitative framework to determine the extent of individual specialization, we can examine how the magnitude of individual specialization may vary across populations. This framework has revealed that among‐individual diet variation is widespread (Bolnick et al., 2003), especially in upper trophic levels (Araujo et al., 2011). Several reviews have extensively reported on the ecological causes (Araujo et al., 2011) and effects (Bolnick et al., 2011) of such individual specialization.

Individual phenotypic variation has as well been studied commonly in relation to adaptive radiation (Schluter, 2000). Such studies have often focused documenting specialization in morphology and/or behavior that can then be related to the harvest of resources (e.g., Bolnick et al., 2003). Such specialization also varies across populations (Svanbäck & Bolnick, 2005; Svanbäck, Eklöv, Fransson, & Holmgren, 2008; Svanbäck & Persson, 2004) but the influence of ecological factors on such variation has not much been studied (but see, Nosil & Reimchen, 2005). There are strong indications that competition may be a key factor for such variation, leading to divergent selection and character release (e.g., Grant & Grant, 2006; Schluter & McPhail, 1992). Furthermore, phenotypic variation within populations is believed to correlate positively with variation in environmental factors (e.g., Hedrick, 1986). Variation in environmental factors creates “ecological opportunity” for population specialization (Nosil & Reimchen, 2005; Stroud & Losos, 2016). When examining individual diet and phenotypic variation, the role of ecological factors (such as habitat characteristics) on the direction and the strength of correlation between diet and morphology of individuals is rarely studied (but see, e.g., Binning & Chapman, 2010). Such a study can help identify important factors for the first steps of population divergence, especially if several allopatric populations are compared.

Northern freshwater fishes are a good candidate for such a study. They inhabit new (available since the last glaciation <14,000 years ago) relatively species‐poor environment, with a number of available niches, and thus “ecological opportunities,” for colonizing fish. The combination of available niches, few competing species, and often high intraspecific competition has resulted in the evolution of great diversity within and among related species (e.g., Schluter, 2000; Snorrason & Skúlason, 2004). This diversity can be connected to different resource use and has been termed resource polymorphism (Skúlason & Smith, 1995). Arctic charr (*Salvelinus alpinus*) displays an extensive phenotypic variability within and across populations, throughout its range (Klemetsen, 2010; Kristjánsson et al., 2011; Noakes, 2010; Skúlason, Antonsson, Guðbergsson, Malmquist, & Snorrason, 1992). An interesting aspect of the observed diversity of Arctic charr is the common occurrence of a small benthic morph in well‐defined habitats (Klemetsen, 2010; Kristjánsson, Skúlason, Snorrason, & Noakes, 2012).

In Iceland, small benthic charr populations can be found within the volcanic active zone, where they inhabit springs within lava fields (Kristjánsson et al., 2012; Sigursteinsdóttir & Kristjánsson, 2005; Snorrason & Skúlason, 2004; Sturlaugsson, Jónsson, Stefánsson, & Guðjónsson, 1998). These populations are far derived from their ancestral phenotype (an anadromous charr) and seem to be adapted to the lava and spring habitats they inhabit through paedomorphosis. They are small fish with a deep body, relatively large fins, a subterminal mouth, dark coloration, and persistent parr marks (Kristjánsson et al., 2012). There are clear indications that these populations represent parallel evolution in relation to habitat type and have evolved independently at each locality (Kapralova et al., 2011). Although these populations show a common phenotype in relation to lava and spring habitats, they have retained morphological and diet variation among populations. These variations could be related to the ecological surroundings of the population, especially the associated habitat of the spring, which can flow into either a stream (rheocrene) or a pond (limnocrene) (Kristjánsson et al., 2012). Rheocrene and limnocrene springs where this Arctic charr morph can be found differ in their invertebrate fauna, where invertebrate diversity is lower in rheocrene springs (mainly chironomidae), while increased number of epibenthic crustacean can be found in limocrene springs (Govoni, 2011). This indicates that the total niche width in ponds is likely wider than in streams (Govoni, Kristjánsson and Ólafsson 2017; Kristjánsson, 2008). The stability in invertebrate composition of springs has not been studied in Iceland yet, although physical characteristics of springs are quite stable over time, especially temperature. This has been shown in both Iceland (Kreiling, personal communication) and abroad (e.g., Rosati, Cantonati, Primicerio, & Rossetti, 2014). Arctic charr is the most commonly found fish species in Icelandic cold‐water springs, but in some cases, threespine stickleback (*Gasterosteus aculeatus*) and brown trout (*Salmo trutta*) can be found in low numbers (Kristjánsson, personal observations, 2008).

Measurements of individual specialization have seldom been reported in Arctic charr (e.g., Knudsen, Primicerio, Amundsen, & Klemetsen, 2010). The few studies have systematically compared individual specialization in two contrasting morphs (pelagic vs. limnetic), but no studies in polymorphic fishes have compared individual specialization in a number of allopatric populations of a highly diverged morph. Behavioral studies on Arctic charr have shown that naïve Arctic charr select prey based on their trophic morphology, both in laboratory and in the field (Garduno‐Paz & Adams, 2010), indicating clear individual diet specialization. Such relationships between behavior, morphology, and diet may indicate first stages of population divergence. To get an estimation of whether and to what degree divergence exists within a population, diet overlap can be compared among all individuals and compared to phenotypic (morphology) distance (Bolnick & Paull, 2009). The paper of Bolnick and Paull (2009) has been withdrawn (Bolnick, 2016) because of a misinterpretation of the statistical results. However, we believe the idea and methods set forward there are still valid, and we will refer to the paper here. Such comparison takes its roots in the modeling of niche evolution theory which predicts that among individual competition should drive individuals that differ in morphology further apart in resource use and thus reduce competition among them (Bolnick et al. 2003; Dieckmann & Doebeli, 1999; Rouchgarden, 1972; Slatkin, 1979). It is, however, unknown whether ecological variables play an important role in the first steps of population divergence. This may be seen in clear relationship between morphology and diet and thus increased individual specialization (Svanbäck & Bolnick, 2005, 2007). Ecological variables may influence the strength of the relationships between morphology and died by acting upon both morphological variation, for example, through phenotypic plasticity (West‐Eberhard, 2003), and upon the variation in diet, for example, through changes in invertebrate composition (e.g., Govoni, Kristjánsson & Ólafsson 2017) and thus available prey and/or diet selection (Svanbäck & Persson, 2004). Furthermore, it is unclear how the relationships between morphology and diet are shaped in populations showing strong local adaptations to their habitat, such as the small benthic charr.

Here, we investigate the magnitude of morphological and dietary differences between individuals in 18 populations of small benthic charr in Iceland in relation to ecological variables (habitat characteristics). We included variables that we believe influence the “ecological opportunities” for these populations. Those are variables related to habitat complexity, and thus available microhabitats, and variables that have been shown to influence invertebrate composition in cold‐water springs (e.g., Govoni, Kristjánsson & Ólafsson 2017). Although all these variables have the potential to influence the strength of the relationships between morphology and diet, we specifically predict that we will see stronger relationships in populations in limnocrene springs than in populations in rheocrene springs. This prediction is based on the different nature of these two spring types, where limnocrene springs have more diverse invertebrate composition (Govoni, Kristjánsson & Ólafsson 2017) reflected in more diverse diet of small benthic charr (Kristjánsson et al., 2012).

## 2 METHODS

Small benthic charr were collected from 18 populations widely distributed across Iceland (Kristjánsson et al., 2012). The sampling took place in the years 2004–2006, and each population was visited once during the summer months (June, July, or August). The temporal distribution of sampling locations was due to logistic reasons, but it was decided to sample only during the most productive months of the year to minimize variation. Spring‐fed habitats are known to be stable in physical characteristics, especially in temperature (e.g., Rosati et al., 2014). Eight populations were collected from spring‐fed ponds or lakes and ten populations from spring‐fed streams. At each sampling location, 30–100 fish were collected by electrofishing. In all populations, fish densities were high and all fish were collected within an hour. In all locations, individuals were collected within a small spatial range (<50 m) to reduce variation in resource (see recommendations of Araujo et al., 2011).

Captured fish were euthanized using an overdose of 2‐phenoxyethanol (1 ml/L), and frozen at −20°C. Back in the laboratory, individuals were measured for fork length (to the nearest mm), weighed (nearest 0.1 g), photographed on the left side (Nikon CoolPix 800, 3.2 megapixels), and then dissected for analyses of stomach content. Stomachs were fixed in 5% buffer formalin and then transferred to 70% ethanol before examination.

We randomly selected 30 fish per population for analysis of diet composition. For each stomach sample, invertebrates were counted and identified to the lowest possible taxonomic level under a dissecting microscope (Kristjánsson et al., 2012). The stomach content was classified into 18 groups: Oligochaeta, Nematoda, Acarina, Arachnida, Amphipoda, Copepoda, Ostracoda, Cladocera, Collembola, Tricoptera, Aphidodeia, Chironomidae, Simuliidae, Lepidoptera, Coleoptera larvae, Coleoptera adults, and diptera pupae and flies. The effects of body size were corrected for by calculating within each stomach the proportion of diet groups, from the total number of individuals in stomach. Nineteen fish (i.e., 3.5% of the fish) had empty stomachs, ranging from 0 to 4 within a population, and were removed from subsequent analyses.

Within each population, the average similarity index (IS) was calculated. The IS index is the average of the proportional similarity (PS) index of all individuals in a population (Bolnick, Yang, Fordyce, Davis, & Svanbäck, 2002). The PS index is calculated as follows for each individual:

$$\text{PS}=1-0.5\sum_{j}\left|p_{\mathrm{ij}}-q_{j}\right|,$$

where *p*_*ij*_ is the proportion of individual's *i* diet made up of *j*th food category and *q*_*j*_ is the average proportion of the *j*th food category of all the individuals within the population. This index is an estimation of individual specialization within the populations. A PS value of 1 is given to an individual where the proportion of diet categories is the same as observed for the whole population. As PS approaches 0, there is less overlap between an individual's diet and the diet of the population (Bolnick et al., 2002). The average similarity index was compared between main habitat types using one‐way ANOVA.

Within each population, the pairwise diet similarity (PS_*ij*_) was also calculated between each pair of individuals. PS_*ij*_ is a quantitative measure of the mean diet overlap between individual *i* and individual *j* and was calculated as follows:

$${\text{PS}}_{\mathrm{ij}}=\sum_{k}\min\left(p_{\mathrm{ik}},p_{\mathrm{jk}}\right)$$

where *p*_*ik*_ and *p*_*jk*_ are the proportions of *k*th prey categories in individual *i*'s and *j*'s diet (Bolnick & Paull, 2009; Bolnick et al., 2002; Schoener, 1968). PS_*ij*_ ranges from 1 (when *i* and *j* feed on same preys in same proportions) to 0 (when the two individuals do not share any prey; Bolnick & Paull, 2009; Bolnick et al., 2002). The computation of these PS_*ij*_ resulted into a pairwise diet similarity matrix. Mean IS, prey proportions, and the pairwise diet similarity matrix for each population were calculated using *IndSpec1* package in R (Zaccarelli, Mancinelli, & Bolnick, 2013).

**Figure 1:**
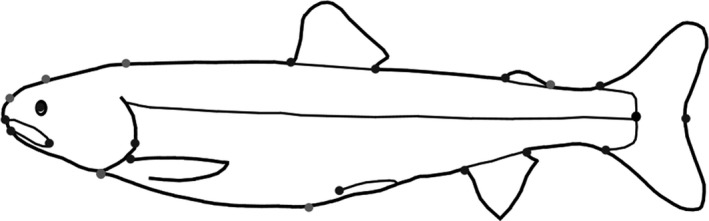
Landmarks used for analysis of morphology of small benthic Arctic charr. Sliding landmarks are shown with light gray dots

Morphological analyses were performed using geometric morphometrics following previously described methods for Arctic charr (Kristjánsson et al., 2012; Sigursteinsdóttir & Kristjánsson, 2005). A detailed description of morphological measurements can be found in Kristjánsson et al. (2012). On each digital image, 22 homologous landmarks were digitized using the software TPS‐dig 2 (Morphometric programs by F. J. Rohlf—http://life.bio.sunysb.edu/morph/). Six of these landmarks were sliding semi landmarks; the other 16 were fixed landmarks (Figure 1). The morphometric data were corrected for up or down bending of the specimens using the “unbend” module in the TPS‐Util program. Relative warp analysis in TPS‐relw was used to analyze for differences in morphology, while controlling for geometric body size. From this analysis, we obtained a weight matrix that was used in further morphometric analysis. Pairwise Procustes distances were calculated among all individuals using the program TPS‐Small, resulting in a morphology distance matrix.

In order to analyze for correlation between the pairwise distance matrices for morphology, each pair of individuals in each population needs to have a PS_*ij*_ value and a morphological distance value. However, because the 30 randomly selected fish for diet analysis were not always suitable for body shape analysis (e.g., low quality of the photographs), diet and morphological distances were calculated on as many fish as available in each population for better accuracy (Table 1). Each matrix was then redesigned and reordered while retaining only the pairs of individuals with both a PS_*ij*_ value and a morphological distance value. The pairwise matrix for morphology and the pairwise matrix for diet were superimposed, and correlation was assessed between them using a Mantel test (Mantel, 1967) with 1,000 replicate permutations. Both steps, the superimposition of the matrices and the Mantel tests, were performed using PASSaGE v.2 software (Rosenberg & Anderson, 2011).

**Table 1:** Correlation between pairwise diet similarity (PS_*ij*_) and pairwise Procustes distances within each population of Icelandic small benthic Arctic charr

Populations	Habitat	*N*	*r*	*p*‐Value
Birkilundur	Pond	30	−.386	.001***
Botnar	Stream	25	−.201	.018**
Grafarlönd	Stream	28	−.332	.001***
Herðubreiðalindir	Stream	28	−.204	.001***
Hlíðarvatn	Pond	30	−.395	.001***
Kaldárbotnar	Pond	31	−.393	.001***
Keldur	Stream	27	−.236	.007**
Klapparós	Stream	29	−.164	.031*
Lækjarbotnar	Stream	30	−.244	.001***
Miðhúsaskógur	Pond	29	−.323	.001***
Mývatn—cave	Pond	26	−.525	.001***
Oddar	Stream	30	−.337	.001***
Presthólar	Stream	29	−.298	.001***
Sílatjörn	Pond	29	−.340	.001***
Silungapollur	Pond	22	−.213	.004***
Skarðslækur	Stream	27	−.372	.002***
Straumsvík	Pond	24	−.242	.001***
Þverá	Stream	27	−.400	.001***

The table shows habitat type, correlation coefficient (*r*), the number of individuals (*N*), and Mantel's test two‐tailed *p*‐values are provided for each population.

Stars refer to levels of significance. *< 0.05, **< 0.01, ***< 0.001

To test for a possible effect of habitat on the strength of the correlation between diet and morphology distances, we performed a *t*‐test on the coefficient of correlation between rheocrene and limnocrene populations. Several physical characteristics were also measured at each sampling location (Table 2): temperature (°C ± 0.1), pH (± 0.1), percentage of rock on the bottom, current velocity (ms^−1^ ± 0.1), conductivity (μs/cm ± 0.1), and bottom complexity. Bottom complexity was assessed using two methods (i.e., the chain and the board), these methods capture the complexity of the bottom in a different way. By laying out a chain of fixed length along the bottom and then measure vertical distance the chain reached. The more complex the bottom, the shorter vertical distance the chain would reach. The second method was by photographing a board, with a number of pins that capture the contour of the bottom. The distance the pins would reach along the board, from the lowest pin was measured, and the variance across all pins calculated. The higher the variance, the more complex the bottom. The methodology for these measurements is further described in Kristjánsson (2008). The correlation coefficient between the diet‐morphology and the physical characteristics was assessed using Pearson's correlations. Statistical analyses were performed using R v. 2.9.2.

**Table 2:** Physical characteristics of 18 spring sites used for a study of relationship between diet and morphology of Icelandic small benthic charr populations

Location	Habitat	Board	Conductivity, years	pH	Temperature, °C	Chain	Proportion rock	Velocity, m/s
Sílatjörn	Pond	4.93	56,000	8.01	5.4	5.75	10	1.66
Hlíðarvatn	Pond	14.80	64	7.60	7.8	3.67	90	0.01
Mývatn—cave	Pond	NA	NA	NA	8.0	4.20	30	0.00
Straumsvík	Pond	9.91	85,000	9.08	5.0	3.15	99	0.01
Birkilundur	Pond	12.96	95	7.59	5.4	3.15	60	0.01
Kaldárbotnar	Pond	5.27	53	8.83	4.5	4.45	90	0.06
Miðhúsaskógur	Pond	7.64	48	9.24	5.5	3.35	70	0.07
Silungapollur	Pond	7.43	72,000	9.43	3.6	4.05	50	0.02
Þverá	Stream	2.56	58,000	7.83	4.9	5.08	95	0.11
Presthólar	Stream	4.46	93	8.24	4.8	4.80	10	0.27
Herðubreiðarlindir	Stream	4.17	134	8.96	5.5	5.11	10	0.10
Lækjarbotnar	Stream	3.87	126	7.33	4.0	5.08	5	5.01
Keldur	Stream	2.79	168	7.91	2.9	4.97	100	0.53
Grafarlönd	Stream	4.08	107	9.38	6.0	4.66	10	0.28
Oddar	Stream	3.86	33	9.79	4.2	4.87	20	0.11
Botnar	Stream	1.97	108	7.96	5.7	4.95	20	27.01
Skarðslækur	Stream	3.16	126,000	7.33	4.0	4.78	20	5.03
Klapparós	Stream	9.12	77	8.34	5.0	3.95	10	0.05

Habitat refers to limnocrene or rheocrene springs, board is a measurement of benthic complexity, chain is another method of benthic complexity.

## 3 RESULTS

**Figure 2:**
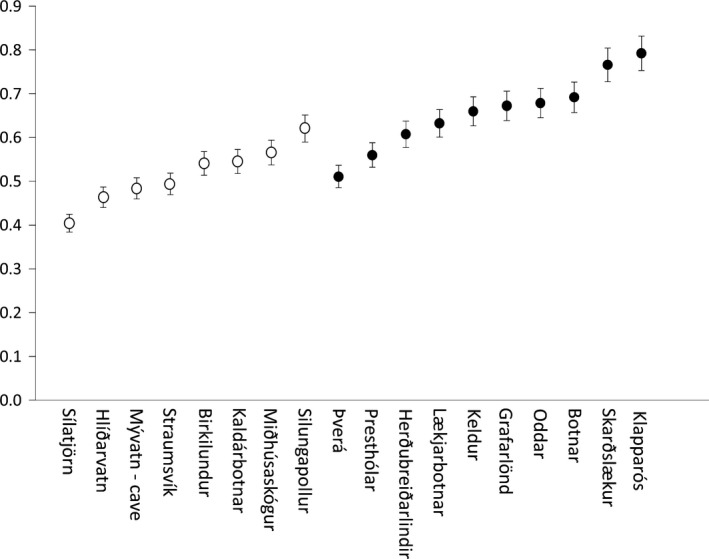
Average similarity index of 18 small benthic charr populations in Iceland, with one standard error. Low scores indicate that there is more individual specialization within the population. Distribution of pond spring (closed circles) and stream spring (open circles) populations are shown

Diet was more variable in ponds than in the streams. Thus, the IS differed between main habitat types (*F*_(1,18)_ = 19.19 *p* < .001), and it was greater in stream (0.66 ± 0.086) than in pond habitat (0.50 ± 0.076; Figure 2). Pairwise diet similarity (PS_*ij*_) ranged from 0.30 to 0.73 and averaged 0.49 ± 0.12 (mean ± *SD*). In some populations, there were indications of bimodal relationships in the distribution (from both limnocrene and rheocrene habitats; Appendix S1). These populations were not the populations having the highest correlation between morphology and diet.

**Figure 3:**
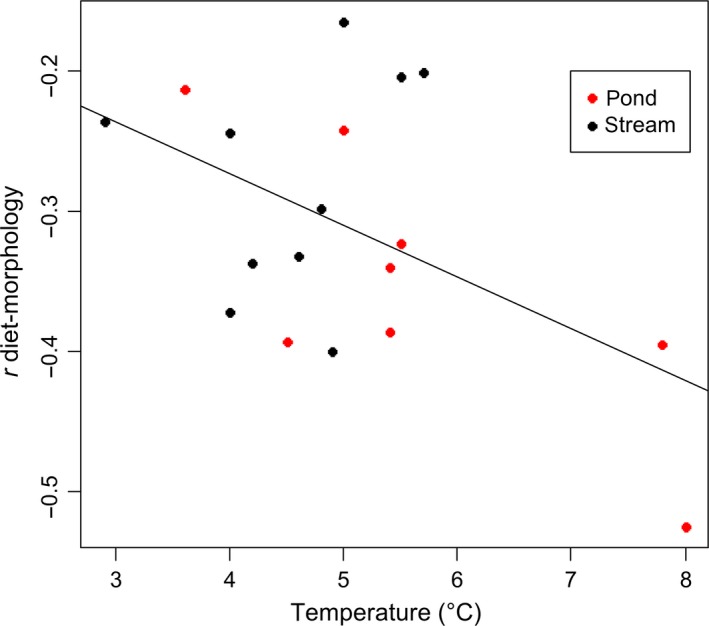
Linear relationship between the temperature and the correlation coefficient diet‐morphology (*r*) in 18 small benthic Arctic charr populations. Each point represents the coefficient correlation between diet and morphology for one population inhabiting a stream (close circles) or a pond (open circle). The solid line represents the correlation between *r* and the temperature regardless of the habitat

There was no clear bimodal relationship in the distribution of morphological distances (Appendix S1). Distances ranged from 0.044 to 0.075 and averaged 0.059 ± 0.008 (mean ± *SD*). In all 18 populations, there was a clear relationship between morphology and diet, where fish of similar morphology ate similar food. This relationship was in the same direction in all populations where PS_*ij*_ was negatively correlated with Procustes morphological distances (Table 1). Morphological distance explained about 2%–27.5% of the total variance in diet similarity among individuals depending on the population. When comparing the two habitat types, the difference in correlation coefficients was close to being significant (*t*‐test: *t*_(18)_ = −1.82, *df* = 18, *p* = .085). In stream habitat, the mean correlation coefficient was −0.28 ± 0.080, whereas it was −0.35 ± 0.091 in pond habitat. Only temperature was negatively correlated with the correlation coefficient (Table 3). The correlation coefficient between diet and morphology (*r*) was lower in habitat with higher temperature (Figure 3). This relationship was mostly driven by two populations (Mývatn cave and Hlíðarvatn) that were warmer than the rest. When these populations were removed, this correlation was not significant (Pearson's correlation, *t*_(14)_ = −0.204, *p* = .84). Other measured physical characteristics of the habitat were not correlated with the correlation coefficient (*r*) (Table 3).

**Table 3:** Results of Pearson's product‐moment correlation between the coefficient of correlation diet/morphological distances within 18 small benthic charr populations in Iceland and the physical characteristics of the habitat

Physical characteristics	*r*	*t*	*df*	*p* Value
Board	−.170	−0.694	14	.515
Chain	.127	0.511	16	.616
% Of rock on the bottom	−.215	−0.088	16	.392
Conductivity	.423	1.807	15	.091
Temperature	−.503	−2.325	16	**.034**
pH	.191	0.752	15	.464
Velocity	.296	1.241	16	.232

## 4 DISCUSSION

Small benthic charr in Iceland have evolved phenotypes that are quite different from the common ancestor, an anadromous charr that colonized Icelandic freshwater systems about 10,000 ya (Kristjánsson et al., 2012). These fish all show signs of paedomorphosis and therefore have retained some phenotypic characteristics commonly found in Arctic charr juveniles. These fish are likely specialized in using the unique habitat of lava springs (Kristjánsson et al., 2012). Within the limits of their habitat (i.e., year around stable cold‐water temperature and structurally restricting), these fish seem to be harvesting all types of available resources (Kristjánsson, 2008; Kristjánsson et al., 2012). We showed that these populations do not represent a randomly mixed collection of generalized individuals, but individuals varying in their level of individual specialization. This can be, for example, seen in the bimodality of the distribution of the pairwise dietary index in some populations. This bimodality may indicate a stronger diet specialization in these populations. This specialization is although not necessarily related to morphology as the relationship between dietary distances and morphological distances was not the strongest in these populations. However, in all the populations, we found clear relationships between morphology and diet, indicating that fish were eating diet best suited to their morphology. We did not get a strong support for our predictions, as we could only see that strength of the relationship between morphology and diet could in some cases be related to temperature, although weakly. This significant correlation was mainly influenced by two populations. Nevertheless, these results suggest that temperature may be an important ecological factor for resource‐related divergence in these populations.

Temperature is a key factor affecting the diversity of invertebrates in the spring habitats where these populations are found (Govoni, Kristjánsson & Ólafsson 2017). Temperature has also been related to diversity in morphology among these populations (Kristjánsson et al., 2012). Increased temperature increases metabolic demands in ectotherms (Gillooly, Brown, West, Savage, & Charnov, 2001), which in turn may result in an increased demand on resource availability and thus competition. Increased competition may, in turn, select for individuals feeding on the prey they are best suited to feed on, and thus, the relationship between morphology and diet becomes stronger (Skúlason & Smith, 1995).

Two populations influenced mostly the connection between temperature and the relationship between morphology and diet. These two populations are both limnocrene springs, where water flow is quite low. Hlíðarvatn is a small pond within a larger lava field in the southern part of Iceland. The location where the charr were caught was variable, mostly rough lava by the edge of the pond with deeper portions >2 m. Charr were mainly caught along the edge, where they were hiding in the lava. The lava caves in Mývatn are unique habitat for small benthic Arctic charr. These caves are commonly partly covered by a lava roof, they open into the groundwater of the area, with no obvious current. Charr were caught along the lava rocks at the openings of these caves. As both of these populations are limnocrene springs, it is highly likely that they have a more complicated invertebrate community structure (Govoni, Kristjánsson & Ólafsson 2017). Higher temperature (and thus higher metabolic demand) and increased opportunities through more diverse available habitats and preys may thus be the key for the observed relationships to be stronger than in other populations.

Although we did not find other ecological factors to affect the relationship between diet and morphology, variables other than temperature may be important in shaping the diversity we observed in this study. Svanbäck and Bolnick (2007) found in an experimental setup with threespine stickleback that increased density of fish, and thus likely increased competition, made individual specialization stronger and further strengthened the relationship between morphology and diet. In this study all small benthic charr were found at high density (Kristjánsson, personal observation, 2008), compared with most other charr populations, but the exact density has, however, not been estimated. High density might be the causal agent for such a clear relationships between diet and morphology seen in all the 18 populations. Differences in fish density might influence the differences we observed among populations. This hypothesis does, however, need to be tested. Furthermore, competition with other fishes is an unlikely causal factor, as although threespine stickleback and brown trout were in some cases observed in the springs, they were always found to be at extremely low density (Kristjánsson, personal observation, 2008).

Previous studies on small benthic charr in Iceland have shown that the spring type seems to be a key factor for observed phenotypic diversity, where fish in limocrene spring habitats had narrower bodies, especially in the caudal area, and had more subterminal mouth when compared to fish from rheocrene spring habitats (Kristjánsson et al., 2012). Here, we show that this is also true for individual diet specialization and the correlation between morphology and diet at the individual level. Habitat type is important for the average diet IS in a population, where diet similarity was higher in stream populations than in pond populations. The correlation coefficient between morphology and diet was close to be significant, and the relationships were stronger in populations where the spring formed a pond or a lake. The stronger individual specialization and the stronger relationships are likely caused by the ecosystem of pond springs being more variable than stream springs (Govoni, Kristjánsson & Ólafsson 2017). Small benthic charr found in ponds usually have a more variable diet, than those found in streams, with crustaceans coming into the diet in addition to chironomid larvae (Kristjánsson et al., 2012).

The observed relationship between morphology and diet shows that there is clear behavioral and ecological diversification within these diverged populations. Such variation between individuals may then further facilitate or promote evolutionary divergence (West‐Eberhard, 2003). Even if all these populations dwell quite diverged small benthic Arctic charr, these populations are clearly at different levels of divergence, perhaps promoted by important similar ecological factors, as discussed above. Whether this diversity is genetic, or caused by plasticity, or a combination of both, this needs further testing. The observed correlation between morphology and diet may be further strengthened by selection and lead to further and faster phenotypic adaptation and evolution (Skúlason & Smith, 1995), especially in population where competition is strong (Svanbäck & Bolnick, 2007). It is, however, important to remember that variation in ecological factors may quickly reverse the observed level of diversification (Seehausen et al., 2008), further showing the importance of taking the dynamic nature of evolutionary and ecological processes into account.

In conclusion, we have demonstrated important and systematic diversity in the relationship between diet and morphology in a high number of small benthic charr populations. These fish are likely adapted to the spring and lava habitat they inhabit and have evolved phenotypes that are quite divergent from the phenotype of their ancestors. A comparison like this one has not previously been reported, where multiple populations have been compared to allow for estimation of the importance of ecological factors for the first steps of divergence, which is seen here as individual specialization. We have, although, to keep in mind that our results represent a single point snapshot of these populations. It is likely, even though spring habitats are environmentally stable, especially in temperature, that there is a temporal (seasonal and annual) variation in available food for these small benthic charr, and thus, the relationship between morphology and diet may be variable. This is currently being tested in some of these populations. Our results show the importance of fine scale comparisons within populations, and how systematic comparisons among populations under parallel evolution can contribute toward our increased understanding of evolutionary and ecological processes.

## CONFLICT OF INTEREST

The authors declare no conflict of interests.

## AUTHOR CONTRIBUTIONS

BKK designed the study, collected the data, participated in data analysis and writing. CAL designed the study, participated in data analysis and writing.
