# Supplementary material for: Variation in the magnitude of morphological and dietary differences between individuals among populations of small benthic Arctic charr in relation to ecological factors
Source: Ecol Evol. 2018 Jan 3;8(3):1573–81. doi: 10.1002/ece3.3761 (PMC5792579; doi:10.1002/ece3.3761)
Supplement: Supplementary file 1 [file ECE3-8-1573-s001.docx]

**Appendix 1** – **Distribution of Pairwise diet similarity, *PSij (left),* and of pairwise Procustes distances (right) from 18 populations of small benthic Arctic charr (*Salvelinus alpinus*) in Iceland.**

| 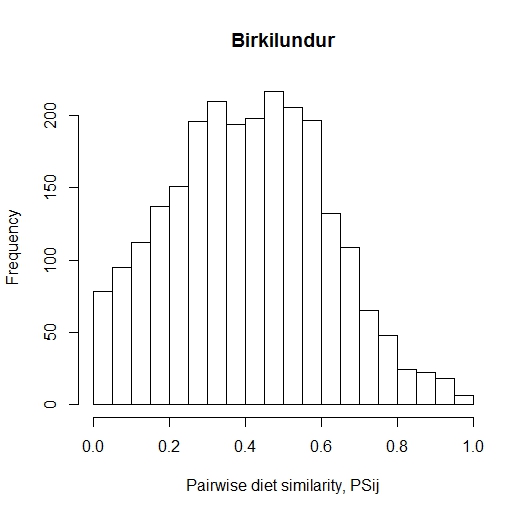 | 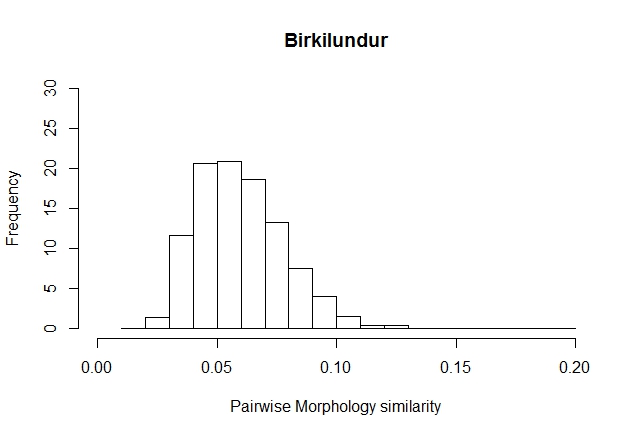 |
| --- | --- |
| 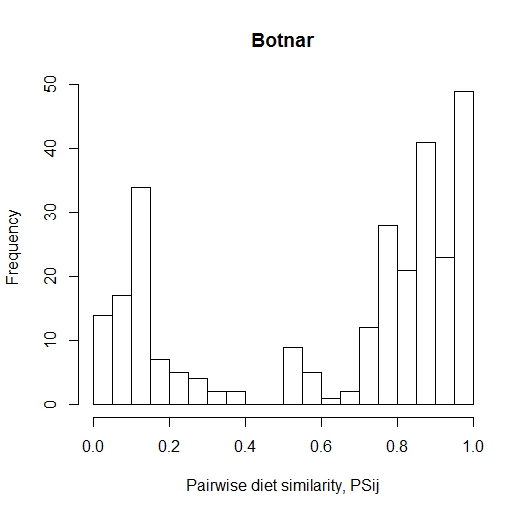 | 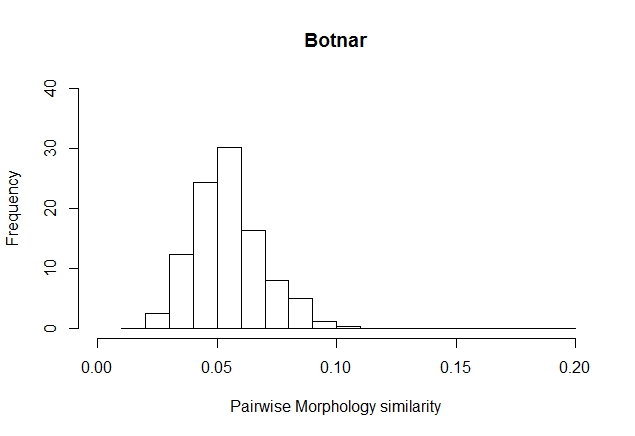 |
| 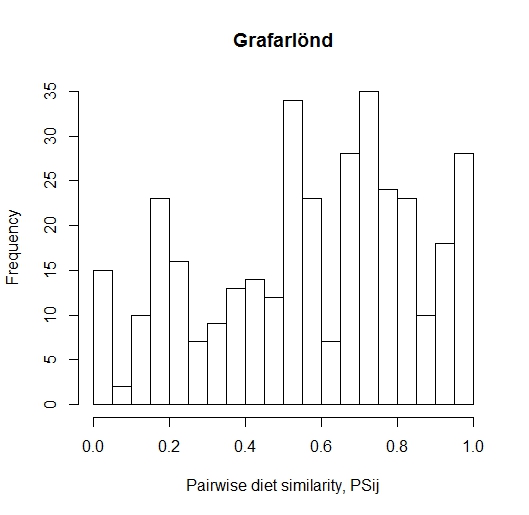 | 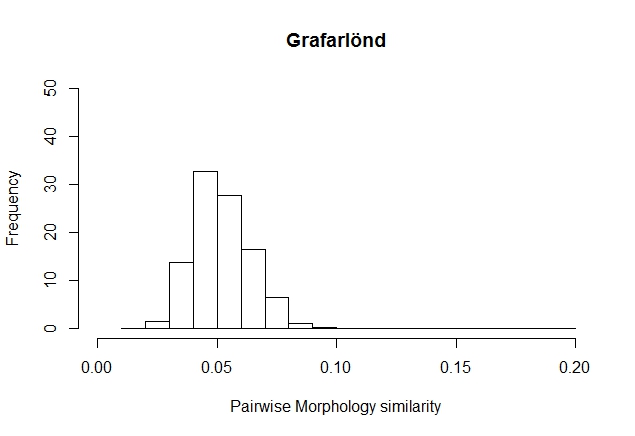 |
| 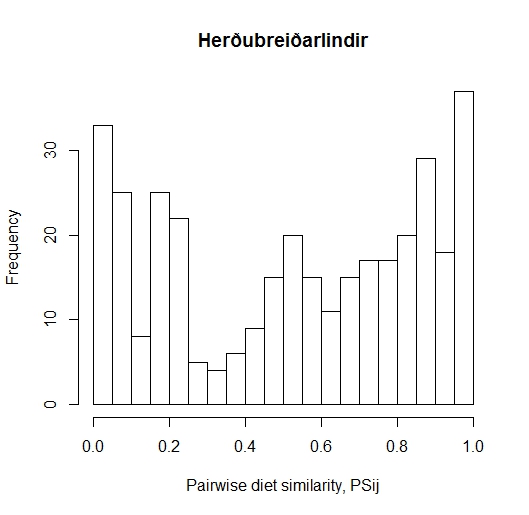 | 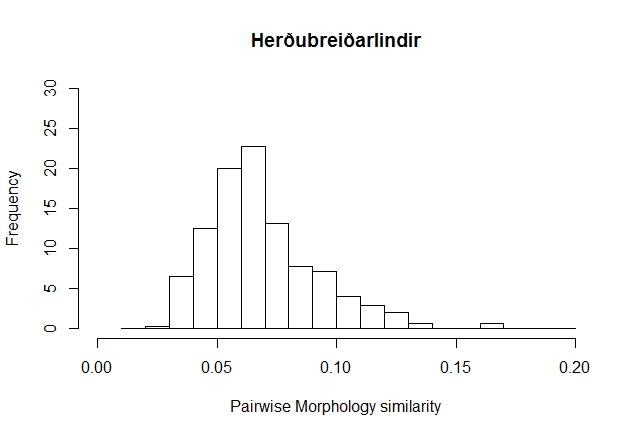 |
| 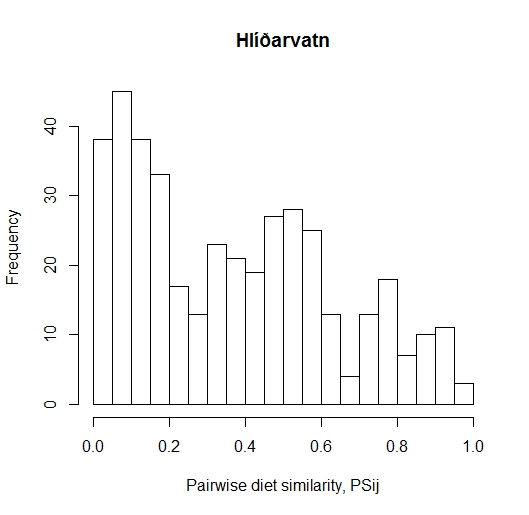 | 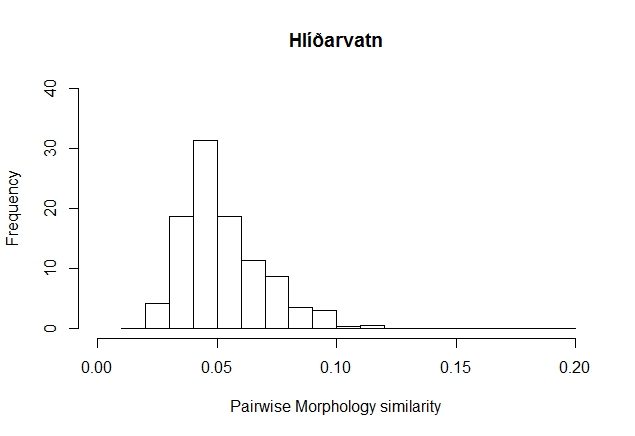 |
| 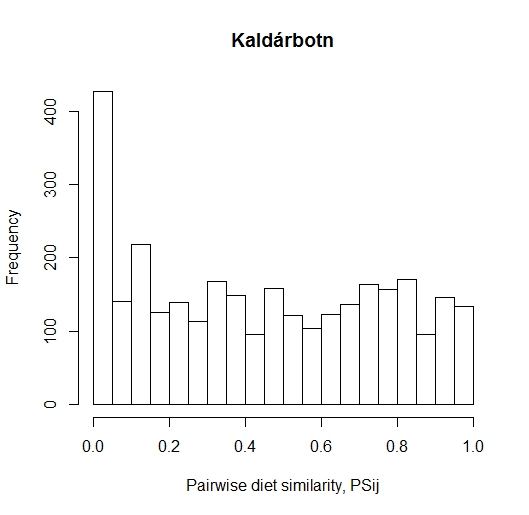 | 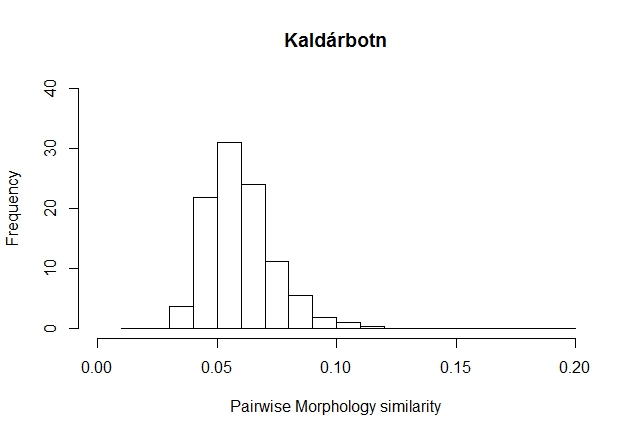 |
| 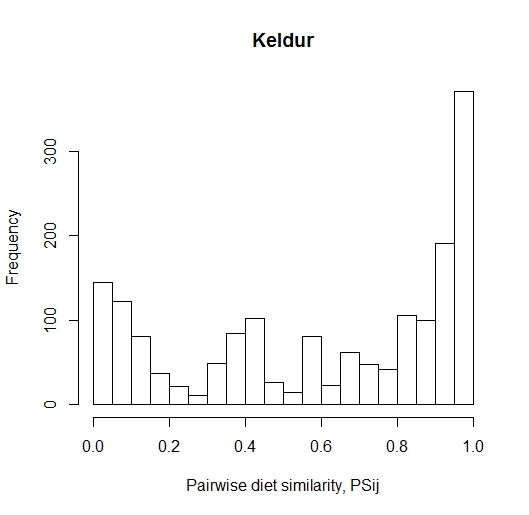 | 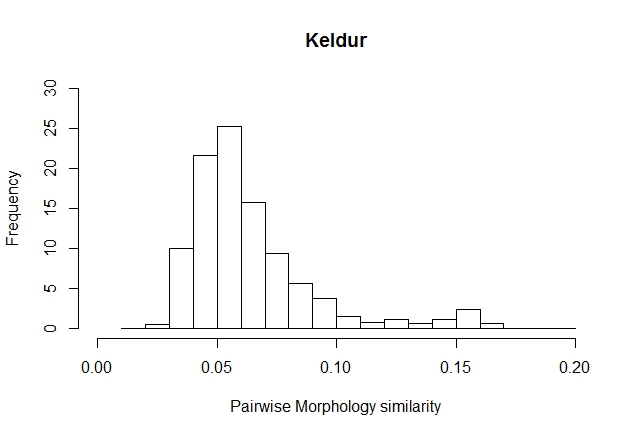 |
| 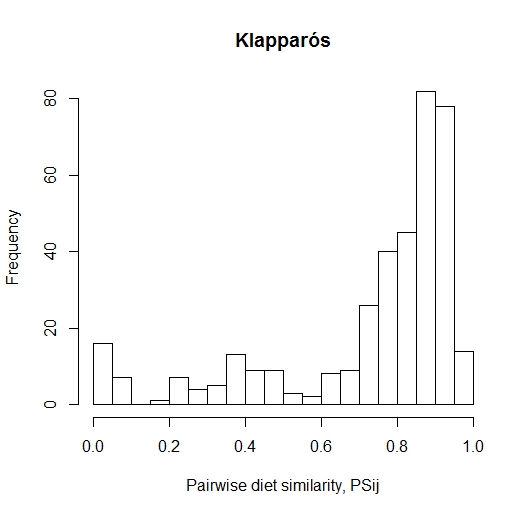 | 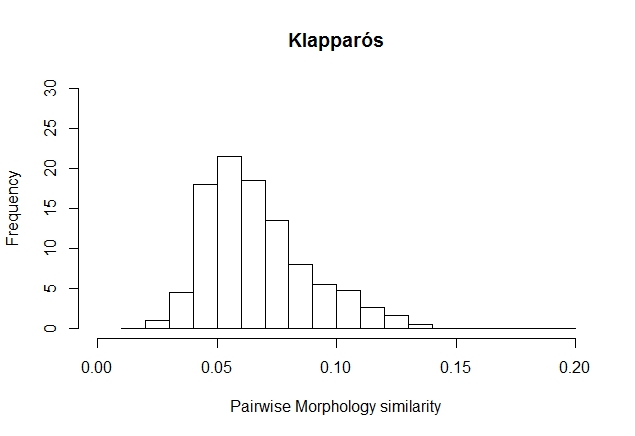 |
| 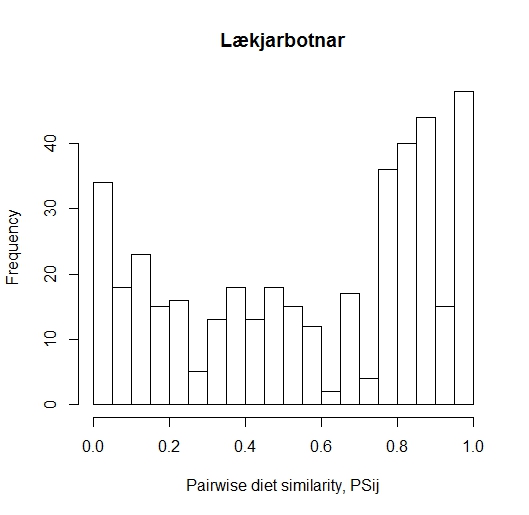 | 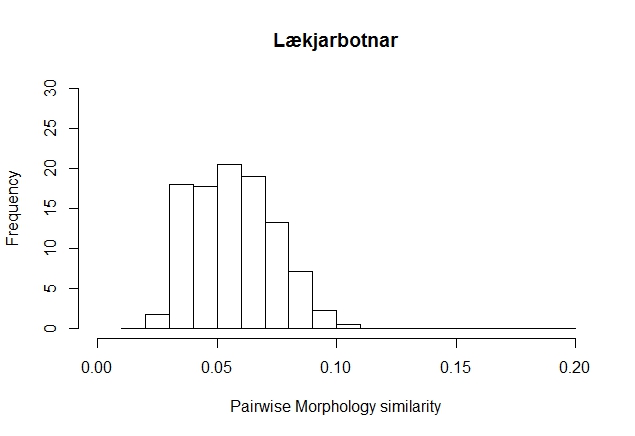 |
| 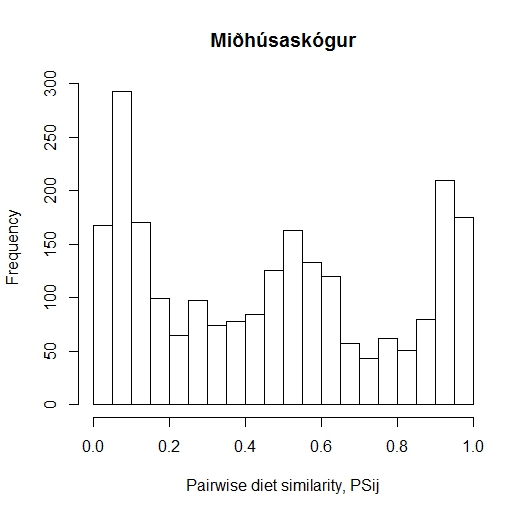 | 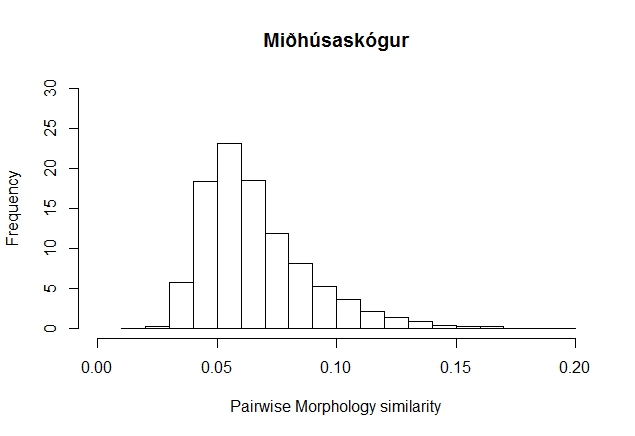 |
| 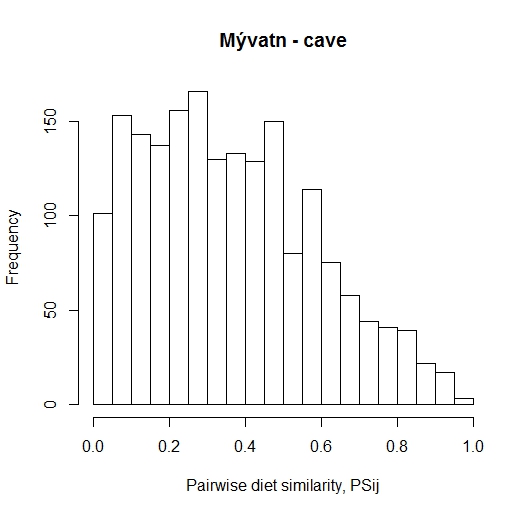 | 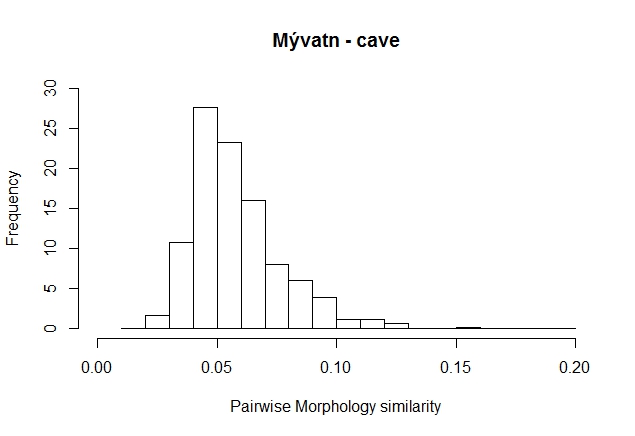 |
| 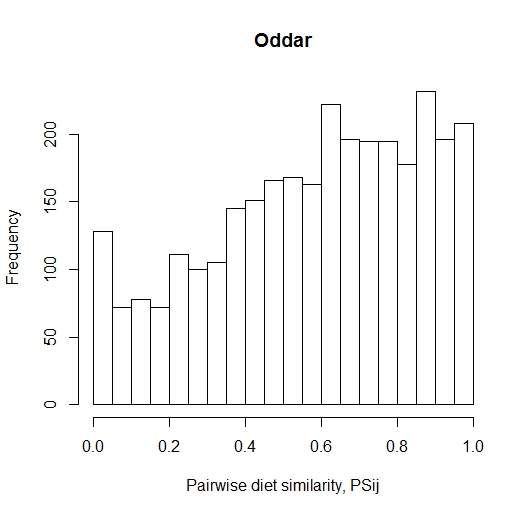 | 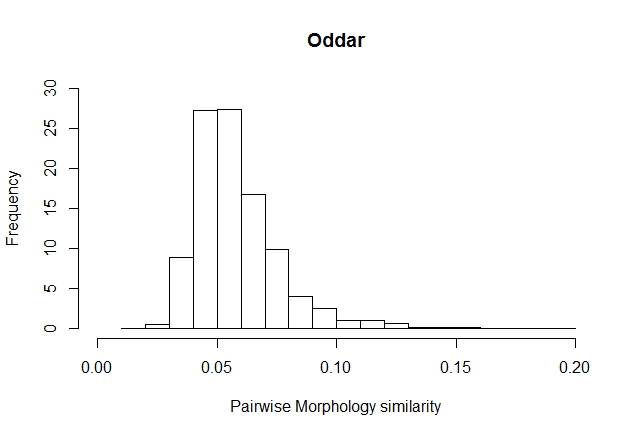 |
| 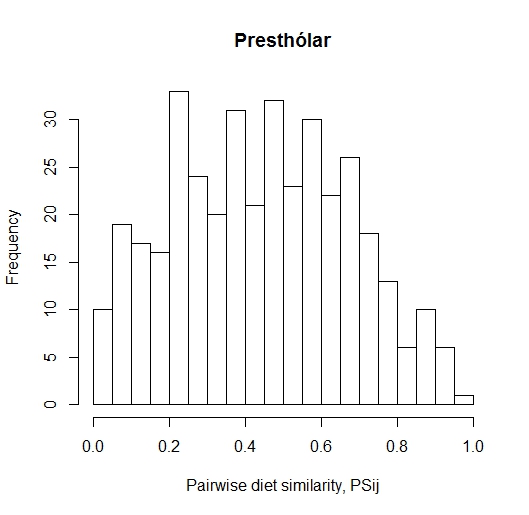 | 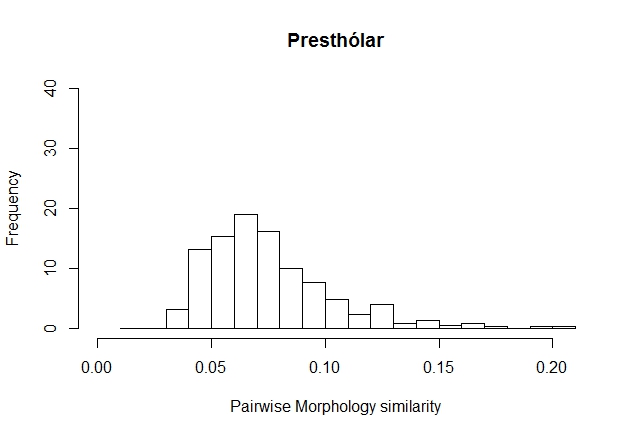 |
| 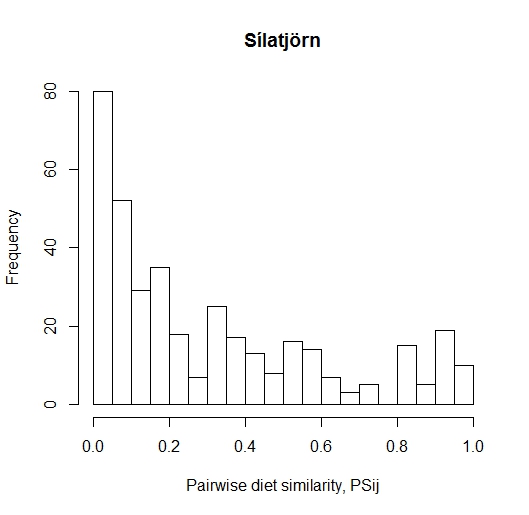 | 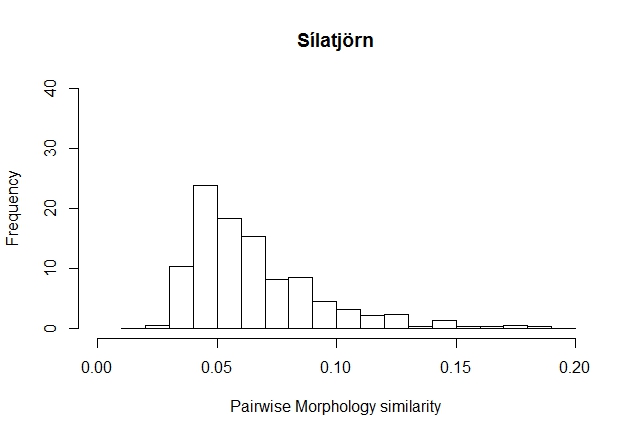 |
| 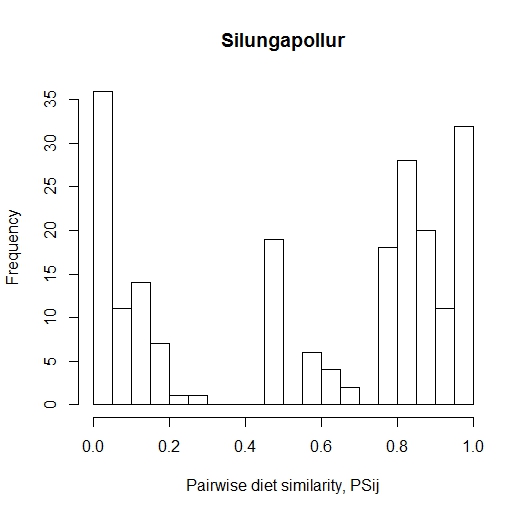 | 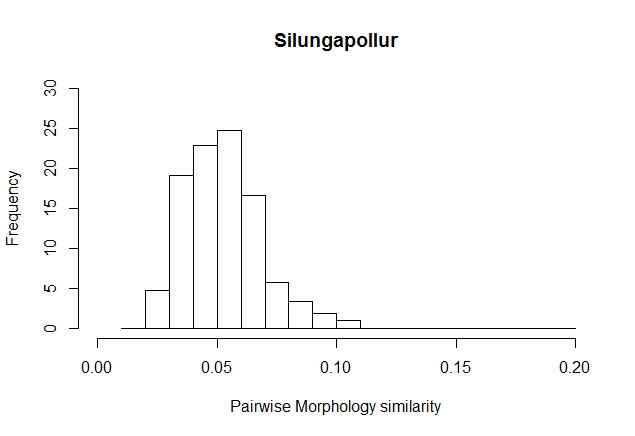 |
| 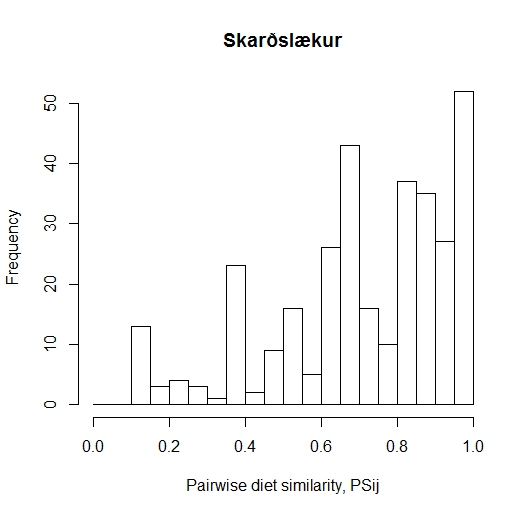 | 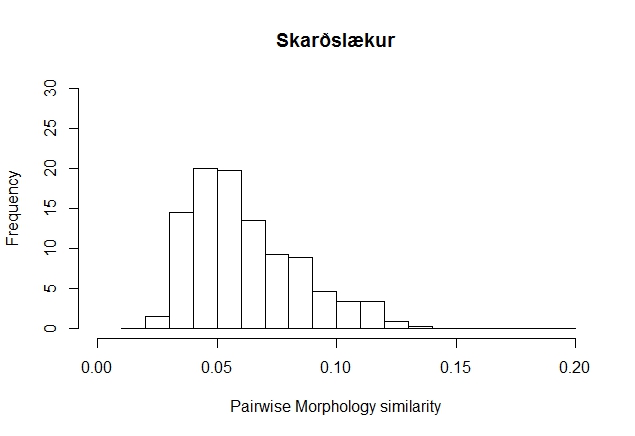 |
| 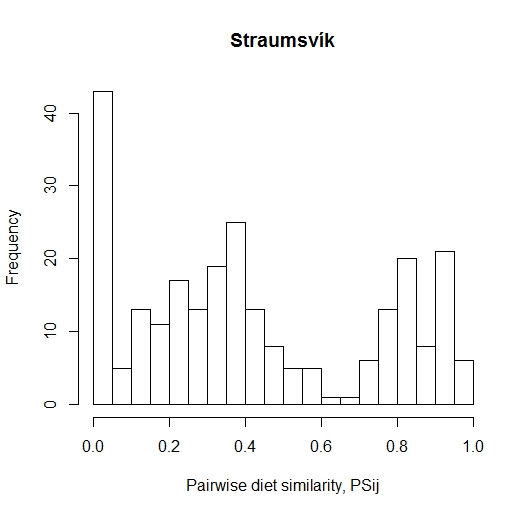 | 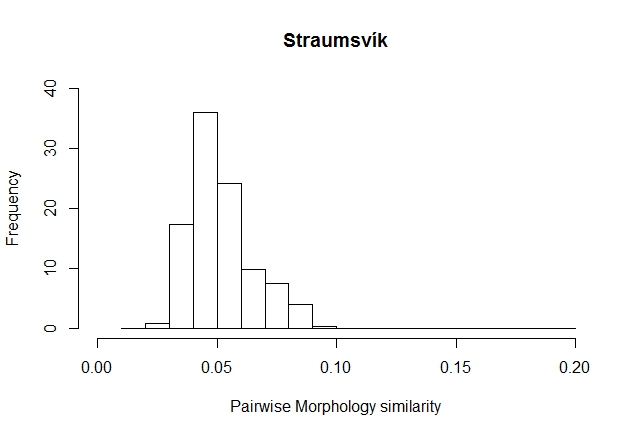 |
| 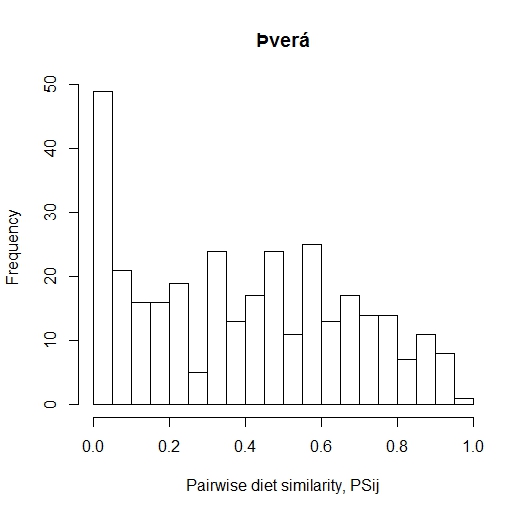 | 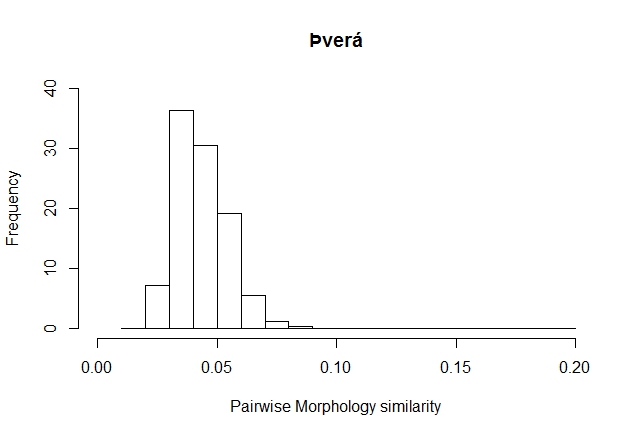 |
